# The Prevention of Rabies

**Published:** 1886-04

**Authors:** 


### The Prevention of Rabies.

On the 26th day of October, 1885, Pasteur communicated to the Academy of Sciences, of France, a method for the prevention of rabies, giving the technique of the procedure, and the results of its application to a single patient, the Alsation shepherd boy.

At that time he offered to treat gratuitously all persons bitten by rabid animals, and retired to the seclusion of his laboratory to await results. His theories awakened a genuine sensation throughout the world; the popular press was filled with extravagant statements, many of which were supposed to come from Pasteur, who really did little but work and wait. He has been lauded by his admirers, criticised by many of his rivals, and ridiculed by some of his German adversaries, who think it impossible that any great discovery can be made by a Frenchman.

But in spite of numerous adverse criticisms, patients flocked into his laboratory from all parts of Europe, and even from America. In ten days from November 1st, ten persons appeared for treatment; in six weeks the number amounted to one hundred, and by February 25, 1886, three hundred and fifty patients had been treated, with but a single unsuccessful case. On the 1st day of March, Pasteur again appeared before the Academy of Sciences, to give the results obtained by his method. In compiling his statistics, the greatest care has been exercised; a certificate from a responsible veterinarian or physician, stating that the dog was really rabid, was required; cases in which the clothing was not torn by the teeth of the dog were rejected, for in such cases, says Pasteur, " no danger was to be feared, for the virus could not enter the tissues, even if the flesh was lacerated." In many cases the dog was obtained, and rabbits inoculated with matter obtained from the nervous centers, thus demonstrating that the animals were really affected with rabies. He gives detailed reports of twenty-five cases who arrived at his laboratory within a period of ten days. In many cases others bitten by the same dog had died. In one case a man presented himself for treatment twenty-seven days after being bitten by a dog which at the same time had bitten two cows and seven hogs, all of which died of hydrophobia, yet this man, four months after, remains perfectly well. Other cases reported are equally interesting. One case, that of Louise Pelletier, a girl ten years of age, resulted fatally. But she was not treated until the thirty-seventh day after being bitten, at which time the period of incubation is past. She was bitten October 3d, presented to Pasteur November 9th, hydrophobia appeared November 27th, resulting in death December 1st. A German pathologist claimed that this death was due to the effects of Pasteur's virus—a possibility which at once presented itself to Pasteur.

Again, to quote from his report, "An important question now presented itself, which virus had produced death, that of the dog, or that of the preventive inoculations? It was easy for me to decide. Twenty-four hours after the death of Louise Pelletier, the cranium was trephined, a small quantity of cerebral matter extracted and inoculated into two rabbits. Eighteen days after, both these rabbits were attacked with rabies almost at the same moment. After their death, virus obtained from their spinal cords was inoculated into other rabbits, who were affected with rabies in fifteen days, whereas, rabbits inoculated with the attenuated virus employed in the treatment of the girl, were affected in seven days at the most."

In the 350 cases treated, no ill effects have been observed; not a single case of erysipelas or abscess, a little red swelling after the last inoculation, and that is all.

In order to ascertain how efficacious his method of prevention has been, Pasteur had recourse to the statistics of rabies, those of Leblanc, the veterinarian, being considered most accurate. From 1878 to 1883, inclusive, 515 persons were bitten by rabid dogs in the Department of the Seine. Of this number ninety-one died of hydrophobia, about one in six, or, accurately, three in seventeen. Granting that the Pellitier case was unsuccessful, we have a mortality of a little more than one-fourth of one per cent. against a mortality of nearly eighteen per cent. where Pasteur's preventive measures have not been employed. By Poisson's method for calculating the limits of error, we may estimate, mathematically, the possibilities and probabilities of error in this matter. The probable error is one per cent.; that is, in successive 350 cases treated, the mortality may vary from seven and one-quarter per cent. to zero. The same formula, applied to Leblanc's statistics, gives a probable error of nearly six per cent.; that is, the mortality of successive 515 cases in which Pasteur's method has not been employed, will vary from twelve to twenty-four per cent.

We may safely conclude, then, that by the employment of Pasteur's method, the mortality from rabies may be reduced eighty-seven per cent., and the distinguished *savant* is justified in saying, "The prevention of rabies after the bite of the animal has been inflicted is an established fact, and a vaccinal establishment for this purpose should be immediately founded. My investigations will not rest here. I would give a strong impulse to the study of other contagious diseases. Many other diseases are analogous to rabies; diphtheria, perhaps, may be treated by the method which I have just expounded."

